# Inverted flap technique versus internal limiting membrane insertion for macular hole in eyes with extremely high myopia

**DOI:** 10.1186/s12886-024-03566-8

**Published:** 2024-07-15

**Authors:** Tsung-Tien Wu, Tzu-Yu Hou, Kai-Ling Peng, Ya-Hsin Kung

**Affiliations:** 1 Department of Ophthalmology, Kaohsiung Veterans General Hospital, 386, Ta-Chung 1st Road, Kaohsiung, 813 Taiwan; 2 School of Medicine, National Yang Ming Chiao Tung University, Taipei, Taiwan, R.O.C.; 3 Shu-Zen Junior College of Medicine and Management, Kaohsiung, Taiwan, R.O.C.

**Keywords:** Axial length, High myopia, Insertion, Internal limiting membrane, Inverted flap, Macular hole, Vitrectomy

## Abstract

**Background:**

To compare the surgical outcomes of the inverted internal limiting membrane (ILM) flap technique and ILM insertion for macular hole (MH) without retinal detachment in eyes with extremely high myopia.

**Methods:**

In this retrospective study, we analyzed 22 eyes with an axial length ≥ 30.0 mm that had underwent MH surgery between April 2015 and August 2021. The surgical procedures involved either an inverted ILM flap or ILM insertion. The outcomes were compared between the two techniques. Closure of the MH was confirmed by optical coherence tomography (OCT). The best-corrected visual acuity (BCVA) was measured before and after surgery. Associated complications were documented.

**Results:**

The median of axial length was 30.64 mm (range, 30.0-34.42). The MH closed in 100% (22/22) eyes and did not recur with a median follow-up of 12.5 months. For the inverted ILM flap technique, the median BCVA improved significantly from 0.80 logarithm of the minimum angle of resolution (logMAR) (range, 0.40-2.00) before surgery to 0.70 logMAR (range, 0.09–1.52) after surgery (*p* = 0.002). In addition, the median of final BCVA was better for the inverted ILM flap than ILM insertion (0.7 logMAR V.S. 1.00 logMAR; *p* = 0.016).

**Conclusions:**

In eyes with extremely high myopia, despite comparable effects on MH closure for both ILM insertion and the inverted ILM flap, the later technique achieved significantly better visual outcomes.

## Introduction

The closure rate of idiopathic full-thickness macular hole (MH) after surgery has reached 90% currently. The surgical outcomes of MH without retinal detachment (RD) in highly myopic eyes are less favorable. The reported MH closure rate varied from 60 to 100% among studies [1–8]. The reason for the worse prognosis may partially be the considerable challenge for manipulation in highly myopic eyes, particularly those with an extremely elongated axial length ≥ 30 mm and severe thinning of the retina. In addition to pars plana vitrectomy, the surgical procedures include peeling of the posterior hyaloid and internal limiting membrane (ILM) to release traction as well as intraocular gas tamponade [7, 8]. In our previous study, despite an enhanced anatomic success of ILM insertion as compared with complete ILM peeling in treating MH in extremely high myopia, the vision did not show obvious improvement [9].

In 2010, Michalewska et al. reported that the inverted ILM flap technique resulted in better functional and anatomic outcomes than complete ILM peeling in treating large idiopathic MH [10]. Later, this technique has been employed to treat MH in different clinical settings. To date, limited studies have compared the inverted ILM flap with ILM insertion in managing MH in eyes with extremely high myopia. The aim of this study was to compare the outcomes of these two techniques in treating MH without RD in eyes with an axial length ≥ 30 mm.

## Methods

The study was approved by the Institutional Review Board of Kaohsiung Veterans General Hospital and was designed in accordance with the Declaration of Helsinki. In this retrospective study, we consecutively recruited eyes with an axial length ≥ 30.0 mm that had undergone MH surgery between April 2015 and August 2021. The patients were followed up for at least three months after surgery. Eyes with an axial length < 30.0 mm, retinal detachment, partial-thickness MH, secondary MH, or a previous history of vitreoretinal surgery were excluded.

A comprehensive ophthalmologic investigation was conducted before and after surgery. The examinations included best-corrected visual acuity (BCVA), refractive errors, axial length, intraocular pressure, slit-lamp biomicroscopy, and indirect ophthalmoscopy. Spectral-domain optical coherence tomography (SD-OCT) (RTVue Scanner; Optovue, Inc, Fremont, CA) was conducted before surgery and at every follow-up visit after surgery. The image was centered on the fovea and a 6.0 mm x 6.0 mm scan was obtained. The MH size was defined as the shortest distance between the edges of the hole, while the measurement was paralleled to the retinal pigment epithelium. The presence of myopic traction macuolpathy, maculoschisis, posterior staphyloma, or chorioretinal atrophy was documented.

All surgeries were performed by the same surgeon (TT Wu). Standard 23- or 25-gauge pars plana vitrectomy, peeling of epiretinal membranes if present, ILM peeling with the assistance of dye staining, followed by 16% or 20% perfluoropropane (C_3_F_8_) gas tamponade were generally performed. Either an inverted ILM flap or ILM insertion was additionally carried out. The ILM insertion technique was adopted prior to 2017, whereas the ILM flap technique was generally employed since 2017. In the ILM insertion technique, ILM was peeled in a circular fashion for approximately 1–2 disc diameters, while the ILM was remained attached at the edge of the MH. Subsequently, the ILM was gently inverted in all directions and inserted into the hole. In the ILM flap technique, the ILM was peeled in the same manner in a circular fashion with the flap attached at the edge of the MH. However, the inferior half of the flap was peeled off, and then the superior half of the flap was gently inverted to cover the surface of the MH. Due to the unavailability of brilliant blue G in Taiwan during that period, 0.05% indocyanine green was used as the dye instead. Meanwhile, we informed all patients of potential ocular toxicity before use.

The subjects were divided into two groups according to the surgical technique. The baseline demographics and surgical outcomes were compared between the groups. Complete MH closure was defined as apposition of the hole edge in all scans on OCT, whilst the retinal layer and foveal depression were restored.

### Statistical analysis

The continuous variables were compared between the groups using Mann-Whitney U test for non-parametric sample. The BCVA was analyzed after the conversion of Snellen to logMAR measurement. The postoperative and preoperative BCVAs were compared using Wilcoxon test. The categorical variables were interpreted using a Chi-square test or Fisher’s exact test. The data were analyzed using IBM SPSS statistical software version 20.0 (Armonk NY). A *p* value less than 0.05 was considered statistically significant.

## Results

A total of 22 eyes from 22 individuals, including 16 (72.7%) women, were consecutively recruited. The median age was 58.5 years (range, 43–69 years). The median of axial length was 30.64 mm (range, 30.0-34.42). The ILM insertion technique was conducted in 8 (36.36%) eyes, and 6 of them had been reported in our previous study [9]. From 2017, the inverted flap technique was conducted in the other 14 (63.64%) eyes. Table 1 summarized the baseline demographics. The two groups were not different in terms of gender distribution, mean age, baseline VA, axial length, lens status, MH size, and presence of posterior staphyloma or schisis.

**Table 1:** Summarizes the baseline characteristics of macular holes in eyes with extremely high myopia

	Total, *N* = 22	ILM insertion *n* = 8	Inverted ILM flap *n* = 14	*P* value
Male, n (%)	6 (27.27)	4 (50)	2 (14.29)	0.137
Age, year (median, range)	58.5 (43–69)	61.5 (51–69)	57 (43–66)	0.082
Axial length, mm (median, range)	30.64 (30.0-34.42)	30.78 (30.16–32.19)	30.64 (30-34.42)	0.815
Phakic, n (%)	9 (40.91)	2 (25)	7(50)	0.380
Size of MH, µm (median, range)				
Minimal diameter	178.41 (45.45–400)	227.27 (45.45-295.45)	162.5(50–400)	0.714
Base diameter	431.82 (100–1875)	392.05 (340.91-1022.72)	568.18 (100–1875)	0.868
Diameter ratio	0.4 (0.05-1.0)	0.5 (0.05–0.73)	0.4 (0.07-1.0)	0.365
Posterior staphyloma, n (%)	22(100)	8 (100)	14 (100)	
Schisis, n (%)	11 (50)	4 (50)	7 (50)	1.000
Baseline BCVA, logMAR (median, range)	1.00 (0.40-2.00)	1.00 (1.00-1.70)	0.80 (0.40-2.00)	0.330

RANGE, standard deviation; MH, macular hole; BCVA: best-corrected visual acuity; logMAR, logarithm of minimum angle of resolution

**Fig. 1:**
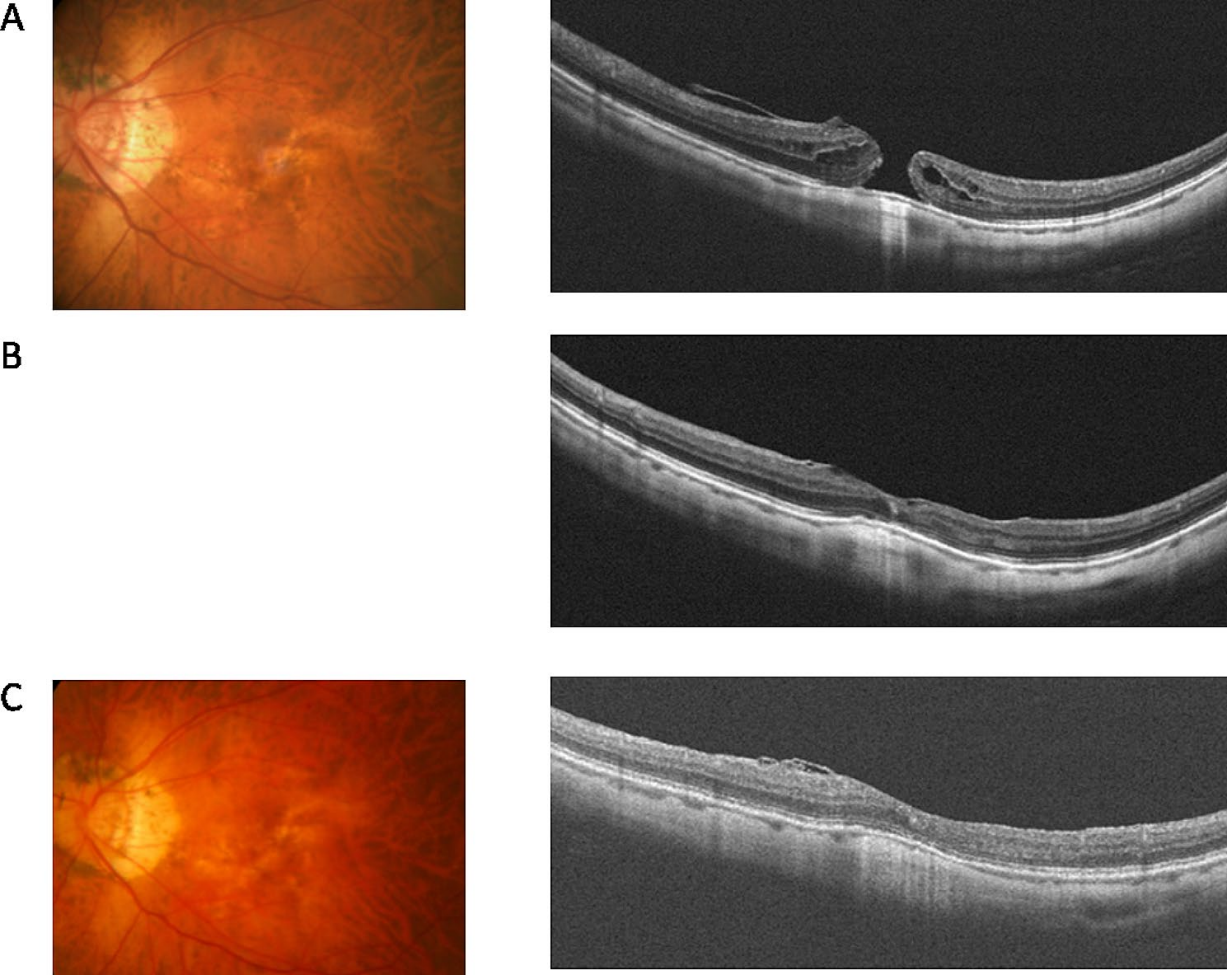
A 61-year-old highly myopic man suffered from progressive blurred vision with metamorphopsia in his left eye for years. The axial length was 30.78 mm. (**A**) Dilated fundus photo and OCT showed a full-thickness macular hole. (**B**) At 2 months after surgery using the inverted ILM flap technique, OCT showed hole closure. (**C**) At one year after surgery, fundus and OCT showed hole closure, and his best-corrected visual acuity was improved from 6/15 to 6/8.6

Table 2 summarized the outcomes of MH surgery. Figure 1 demonstrated the case treated with inverted ILM flap technique. After a median of 12.5-month (range, 3–48 months) follow-up, the MH closed in all eyes, and reopening of the MH did not occur throughout the period. Complications were not observed in any case.

**Table 2:** Shows the surgical outcomes of macular hole repair in eyes with extremely high myopia

	Total, *N* = 22	ILM insertion *n* = 8	Inverted ILM flap *n* = 14	*P* value
Anatomical MH closure, n (%)	22 (100)	8 (100)	14 (100)	
Complication, n (%)	0 (0)	0 (0)	0 (0)	
ELM disruption, n (%)	14 (63.64)	7 (87.5)	7 (50)	0.167
EZ disruption, n (%)	17 (77.27)	8 (100)	9 (64.29)	0.115
V-shaped thin fovea, n (%) BCVA, logMAR (median, range)	6 (27.27)	2 (25)	4 (28.57)	1.000
Baseline	1.00 (0.40-2.00) (SE, 20/200)	1.00 (1.00-1.70) (SE, 20/200)	0.80 (0.40-2.00) (SE, 20/126)	0.330
Final	0.90 (0.09-2.00) (SE, 20/160)	1.00 (0.80-2.00) (SE, 20/200)	0.70 (0.09–1.52) (SE, 20/100)	0.016
*P (baseline versus final)*	0.014	0.715	0.002	
Follow-up time, months (median, range)	12.5 (3–48)	14 (3–48)	10.5 (3–40)	0.441

BCVA, best-corrected visual acuity; ELM, external limiting membrane; EZ, ellipsoid zone; logMAR, logarithm of minimum angle of resolution; MH, macular hole; SD, standard deviation; SE, Snellen equivalent;

In general, the median of logMAR VA improved significantly from 1.00 (range, 0.40-2.00) before surgery to 0.90 (range, 0.09-2.00) at the end of follow up (*p* = 0.014). Among all studied eyes, 86.36% (19/22) presented with improved or stabilized vision after surgery. The vision dropped in two eyes treated with ILM insertion and in one case treated with inverted ILM technique because of the extensive chorio-retinal atrophy over the macula. Even though, the atrophy did not cause significant visual impact in both groups. Rather, the median of BCVA in eyes treated with the inverted ILM flap improved significantly from 0.80 logMAR (range, 0.40-2.00) to 0.70 logMAR (range, 0.09–1.52) after surgery (*p* = 0.002). Meanwhile, the inverted ILM flap appeared to have a better final BCVA than ILM insertion (median, 0.70 V.S. 1.00 logMAR; *p* = 0.016).

## Discussion

Surgical repair of MH is exactly more challenging and complex in highly myopic eyes than eyes with emmetropia. Certainly, the abnormally thin retina, elongated axial length, and chorioretinal atrophy lead to poor visualization and difficult ILM peeling during surgery. Additional factors may probably affect the anatomic and visual outcomes. In fact, progressive elongation of the axial length, chorioretinal atrophy, pathologic thinning and relatively shortened retina may inhibit MH closure, despite complete ILM peeling and successful release of the tangential traction force.

In 2010, Michlewskaet al. introduced an inverted ILM flap technique for large MH [10]. Subsequently, the method became an effective alternative to treat MH especially in difficult cases. In 2014, Michlewska et al. reported a high MH closure rate as 100% in 19 myopic eyes, including six with an axial length ≥ 30 mm, treated with the inverted ILM flap [11]. They believed not only an enhanced proliferation of glial cells from the inverted ILM flap that promoted MH closure but direct reapposition of the photoreceptors to the fovea [10]. Rizzo et al. confirmed the role of the inverted ILM flap as a scaffold for the proliferation of glial cells to facilitate MH closure [12]. In 2017, Dr. Chen modified the technique and introduced a large semicircular inverted ILM flap to cover MH in 17 myopic eyes, among which 11 eyes (64.7%) had an axial length > 30 mm [13]. It turned out to be a 100% MH closure rate after a single operation [13]. Subsequently, Rizzo et al. evaluated MH in eyes with an axial length ≥ 26 mm treated with ILM insertion [12]. The ILM inside the vascular arcades was peeled sparing the fovea, resulting in a ring-shaped ILM island surrounding the MH [12]. Eventually, the ILM was inverted and inserted into the MH. A higher closure rate was achieved by the ILM insertion technique as compared with ILM peeling alone (88.4% vs. 38.9%) [12]. The result was consistent with another study by Bové Álvarez et al. comparing ILM peeling alone and ILM insertion in myopic eyes [14]. The closure rate was 81.2% and 91.7% respectively [14]. Overall, an inverted ILM, either covering or insertion into the MH, provided a more favorable anatomic outcome than complete ILM peeling in myopic eyes. Moreover, the semicircular inverted ILM flap seemed to be superior to ILM insertion as in our study, especially in eyes with extremely high myopia which had an axial length > 30 mm.

The logMAR BCVA was 0.69 at postoperative six months following MH surgery using an inverted ILM flap reported by Dr. Chen [13]. In the study by Rizzo et al. [12]. , the logMAR BCVA was 0.52 and 0.43 nine months after the ILM peeling technique and the ILM insertion technique, respectively. Later, Bové Álvarez et al. demonstrated that the median VA was 0.25 logMAR after 18-month follow-up in the ILM peeling group, while 9.8 months after surgery using ILM insertion, the median VA was 0.40 logMAR [14]. The results were variable among studies. This was probably due to different baseline VA, inconsistent axial lengths, and diverse surgical manipulations.

In our previous study on 14 eyes with MH and a mean axial length of 30.69 mm, the primary closure rate was 37.5% in the ILM peeling group and 100% in the ILM insertion group [9]. Although ILM insertion achieved a significantly higher anatomic success rate, the visual outcome seemed less favorable. The mean BCVA was 1.08 logMAR at 8.5-month follow-up in the ILM insertion group, while 23.9 months after surgery with complete ILM peeling, the mean BCVA remained 0.67 logMAR. Some believed that manipulation of the ILM with insertion of the ILM into the MH, even gently, might injure the retinal pigment epithelium and foveal microstructures and that the inserted ILM may prevent the photoreceptors of MH edges from reapposition. In our study, amorphous tissues from the inserted ILM and consequent defects of the external limiting membrane (ELM) and ellipsoid zone (EZ) at the fovea were demonstrated on the OCT image. As a result, restoration of the foveal structure may be inhibited, resulting in the non-favorable visual outcome.

Mete et al. had treated 70 eyes with axial length > 26.5 mm and macular hole with either complete ILM removal or inverted ILM flap technique, and they reported inverted ILM flap technique achieved a more favorable anatomic success, with 22 times higher success rate compared with complete ILM removal, regardless of the myopic MH diameter [15].

Besides, in 2019, Park et al. had compared ILM insertion and the inverted ILM flap for large idiopathic MH [16]. Though similar effects for MH closure, the EZ recovered better in the inverted ILM flap technique, which may also be associated with a better postoperative BCVA.

However, few studies evaluated ILM insertion and the inverted ILM flap to treat MH in eyes with extremely high myopia. In the present study, we compared the anatomic and functional outcomes of these two techniques to treat MH in highly myopic eyes. In the inverted ILM flap group, the superior ILM flap was inverted inferiorly to cover the MH. Any manipulation was avoided to minimize the mechanical damage. After a single operation, both the techniques achieved a 100% primary closure rate. Recurrence of MH did not occur throughout the follow-up period.

Insertion of the ILM secured the tissue in the MH and directly connected the edges of the hole, while the inverted ILM flap covered and bridged the hole, thereby providing a scaffold to facilitate attachment of the hole edges without interfering with the foveal microstructure. In this study, we found there were 87% of external limiting membrane (ELM) disruption and 100% of ellipsoid zone (EZ) disruption in the group of ILM insertion after operation, and these findings were higher than those in the group of inverted ILM flap (50% and 64.29%, respectively). However, these were not statistically significant, probably due to small sample sizes. Nonetheless, the percentages of V-shaped thin fovea were similar in both groups.

In addition to significantly visual improvement following the inverted ILM flap technique (the median BCVA from 0.80 logMAR to 0.70 logMAR; *p* = 0.002), the median of postoperative BCVA was better for the inverted ILM flap than for ILM insertion (0.70 logMAR V.S. 1.00 logMAR; *p* = 0.016).

In a study by Qi et al. [17], they found that preoperative hole diameter ratio is a predictor for anatomical outcomes of stage III or IV idiopathic macular holes, and the minimum to maximum diameter ratio < 0.6 is predictive for a significantly higher closure rate. We also analyzed our cases and found the medians of hole diameter ratio were < 0.6 in both groups, and this probably contributed to the overwhelming closure rate in our study.

Considering that foveoschisis might influence the outcomes, we subdivided the eyes according to the presence of foveoschisis into two groups and analyzed the outcomes. There was no significant difference between the groups with and without foveoschisis, in terms of ELM disruption, EZ disruption, V-shaped thin fovea, baseline BCVA and final BCVA.

For eyes treated with inverted ILM flap technique, foveoschisis did not affect the anatomic outcomes, and both groups had significant visual improvement from baseline (*p* = 0.042 and *p* = 0.026, respectively). However, eyes with foveoschisis seemed to have a worse post-op BCVA than non-foveoschisis eyes, with the median BCVA of 1.00 logMAR (range, 0.09–1.52) versus 0.40 logMAR (range, 0.15–0.7), but this was not statistically significant (*p* = 0.053).

Limitations of this retrospective study included the small sample size, inconsistent follow-up time, and lack of standardized BCVA measurement. The sampling bias could, however, be minimized by factors including the same operator who did all the surgeries in a single medical center. To the best of our knowledge, this study recruited the largest number of eyes of MH with an axial length ≥ 30 mm.

In conclusion, both ILM insertion and the inverted ILM flap are effective for MH closure in eyes with an axial length ≥ 30 mm. Meanwhile, the inverted ILM flap technique could not only improve the vision but achieve a better final BCVA as compared with ILM insertion. Further prospective randomized trials are necessary to evaluate the anatomic and functional outcomes of MH surgeries in eyes with an extremely elongated axial length.

## Data Availability

No datasets were generated or analysed during the current study.
